# Development and Initial Validation of the Chinese Version of the Florida Surgical Questionnaire for Parkinson's Disease

**DOI:** 10.1155/2020/8811435

**Published:** 2020-12-12

**Authors:** Tao Wang, Yiwang Zhang, Yixin Pan, Linbin Wang, Chencheng Zhang, Jun Liu, Liuguan Bian, Bomin Sun, Dianyou Li

**Affiliations:** ^1^Department of Neurosurgery, Ruijin Hospital Affiliated to Shanghai Jiaotong University School of Medicine, No. 197 Ruijin Second Road, Huangpu District, Shanghai 200025, China; ^2^Center for Functional Neurosurgery, Ruijin Hospital Affiliated to Shanghai Jiaotong University School of Medicine, No. 197 Ruijin Second Road, Huangpu District, Shanghai 200025, China; ^3^Department of Neurosurgery, 910th Hospital of People's Liberation Army, No. 180 Huayuan Road, Fengze District, Quanzhou, Fujian 362000, China; ^4^Department of Neurology, Ruijin Hospital Affiliated to Shanghai Jiaotong University School of Medicine, No. 197 Ruijin Second Road, Huangpu District, Shanghai 200025, China

## Abstract

**Background:**

Deep brain stimulation (DBS) for Parkinson's disease (PD) has evolved as a well-established treatment in neurosurgery, and identifying appropriate surgical candidates could contribute to better DBS outcomes. The Florida Surgical Questionnaire for Parkinson Disease (FLASQ-PD) is a reasonable screening tool for assessing DBS candidacy in PD patients; however, a Chinese version of FLASQ-PD is needed for functional neurosurgery units in China. In this study, we translated the FLASQ-PD to Chinese and assessed its reliability and validity for Chinese PD patients.

**Methods:**

The FLASQ-PD was translated before the study formally started. A single-center retrospective analysis of FLASQ-PD was performed at the Ruijin Hospital, affiliated with Shanghai Jiaotong University School of Medicine, between July and December 2019. The Unified Parkinson Disease Rating Scale III (UPDRS-III) was also used to assess PD patients on and off medication. All patients were evaluated for surgical candidacy by specialists.

**Results:**

Overall, 115 PD patients, 25 with parkinsonism and six with multiple system atrophy were consecutively included. Internal consistency of the Chinese FLASQ-PD was roughly adequate (Cronbach's alpha = 0.664). There were significant differences in mean total scores of the Chinese FLASQ-PD between the diagnostic (Kruskal–Wallis H value = 37.450, *p* ≤ 0.001) and surgery-candidacy groups (*H* = 48.352, *p* ≤ 0.001). Drug improvements in UPDRS-III scores were mildly correlated with the Chinese FLASQ-PD scores in the surgery-ready group (Pearson correlation = 0.399, *p*=0.001).

**Conclusions:**

The Chinese FLASQ-PD, which is a simple and efficient screening tool for clinicians, was developed and initially validated in this retrospective single-center study.

## 1. Introduction

Deep brain stimulation (DBS) for Parkinson's disease (PD) has evolved as a well-established treatment in neurosurgery over the last four decades [1]. In 2020, Zhang et al. reported a 43% improvement in Unified Parkinson Disease Rating Scale III (UPDRS-III) scores in PD patients in the off-medication/on-stimulation state during follow-up with combined unilateral subthalamic nucleus (STN) and contralateral globus pallidus interna (GPi) DBS [2]. In the same year, Tsuboi et al. also reported a 29% improvement in the same condition with bilateral GPi DBS at the 1-year follow-up [3]. However, in recent years, studies worldwide still report their primary outcomes (e.g., UPDRS-III, the Parkinson's Disease Questionnaire, levodopa equivalent dose reduction) differently, and even the outcomes of patients differ [4, 5].

So far, several factors have been considered to be potentially associated with the differing effects of DBS. Structural profiles of patients' anatomy such as thalamic and ventricular volumes may predict motor outcomes after DBS, although another study found that the structural and functional connectivity were independent predictors of clinical improvement of STN DBS [6, 7]. However, a conclusion has not yet been drawn regarding the risk factors or predictors of DBS in PD patients. Regardless of individual variance in severity of PD symptoms, identifying appropriate surgical candidates could contribute to the difference in the results in PD patients as well. Therefore, the inclusion criteria of PD-DBS studies serve as a predefined filter for appropriate surgical candidates. Cautiously selecting surgical candidates helps to exclude non-PD patients and also avoid unsatisfactory outcomes after DBS such that both patients and clinicians have confidence during follow-up [8]. The Core Assessment Program for Intracerebral Transplantations (CAPIT) was published in 1992, providing the minimal requirements for a common patient evaluation protocol. However, the program was thought to be too laborious to carry out in large scale trials [9].

To date, two efficient decision-making tools have been developed to screen PD patients and identify surgical candidates for DBS: the Florida Surgical Questionnaire for Parkinson Disease (FLASQ-PD) and the Stimulus tool [10, 11]. Although Chou et al. found FLASQ-PD and Stimulus to be reasonable screening tools for assessing DBS candidacy in PD patients, Coleman et al. suggested that the Stimulus decision tool was a better screening measure to assess DBS candidacy in PD patients than the FLASQ-PD [12, 13]. Comprehensive utilization of these scales in practice may improve the quality and appropriateness of DBS referrals for PD patients and may also simplify the identification of these patients in newly developed functional centers.

However, both tools were originally in English, which limited their applications in countries where English is not the native language. During the past decade, DBS for PD patients has gradually became prevalent in neurosurgery in China [14]. As functional neurosurgery units expanded in China, the number of DBS surgeries varied among units. Whether a PD patient was an appropriate candidate for DBS was still a decision that new units struggled to assess. In particular, tools developed in recent years should be cautious in determining the appropriateness of DBS for PD patients, instead of repeating old errors from other functional neurosurgery centers. A simple decision-making tool prior to DBS treatment should be used as a quick filter to determine the appropriateness of surgical candidates. Therefore, in this study, we translated the FLASQ-PD into a Chinese version and assessed its reliability and validity to develop a general selector specific to Chinese PD patients and make referral easy to generalize in functional neurosurgery units across China.

## 2. Materials and Methods

### 2.1. Translation of English FLASQ-PD

Okun et al. developed and initially validated the original FLASQ-PD in 2003 [10]. We obtained informed permission from his team, and two colleagues independently metaphrased the scale into Chinese (LBW and CCZ). Back-translation (YWZ) was also sent to Okun's team to reconfirm the accuracy of our translation before this study. This study was conducted in accordance with the Declaration of Helsinki and the use of anonymized patient data was approved by the Ethics Committee of Ruijin Hospital, affiliated with Shanghai Jiaotong University School of Medicine.

### 2.2. Contents of Chinese FLASQ-PD

The Chinese FLASQ-PD was a five-section questionnaire (see Supplementary material) with the same content as the English version that included the diagnosis of primary PD or not (Section A), potential contraindications for DBS surgery (Section B), general patient characteristics (Section C), favorable/unfavorable characteristics for DBS surgery (Section D), and medication information subscores (Section E). The scoring system was designed to assign a higher score to a better surgical candidate. Since all items in the questionnaire contributed to the final appropriateness of surgical candidates, and to simplify the scoring method, instead of setting red flags in Section B like the English version, this subscore was calculated inversely (item “1” equaled score zero and item “N/A” equaled score one) and added to the total score. All other subscores used the same scoring method as the original version (i.e., each score in an item was added). Therefore, the possible score range of the Chinese FLASQ-PD was 0–42, and the minimum interval of the scores was 1.

### 2.3. Patient Evaluation

This study was a single-center retrospective analysis performed at the Ruijin Hospital, affiliated with Shanghai Jiaotong University School of Medicine from June 4th to December 31st, 2019. The Departments of Functional Neurosurgery and Neurology collaborated for both patient evaluation and treatment of movement disorders characterized by parkinsonian symptoms. The Chinese FLASQ-PD was assessed by a colleague (TW) for a consecutive series of patients and rechecked by another colleague (LX) independently. The UPDRS-III (off medication for 12 hours and then reevaluated on medication, if available) was assessed in both on and off medication states (if applicable). Two colleagues (LX and YH) independently video-recorded patients, blindly measured UPDRS-III scores, and double-checked each other's final scores.

**Figure 1:**
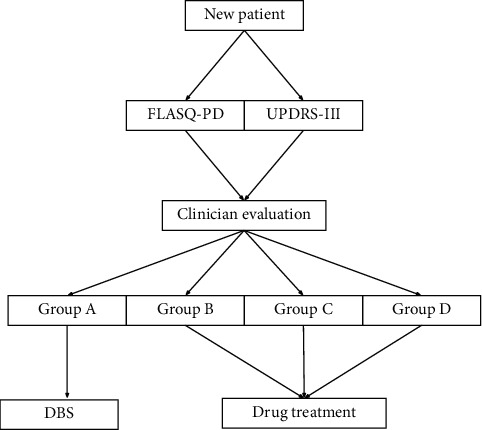
Workflow of patient evaluation and related treatments.

All included patients were reviewed by two clinicians (DYL and JL) specializing in movement disorders and clinically categorized into four groups: Group A, idiopathic PD and ready for surgery; Group B, idiopathic PD and potentially ready for surgery in the future; Group C, idiopathic PD but never applicable for surgery; and Group D, neither idiopathic PD nor a candidate for DBS surgery. DBS surgery would be performed at an appropriate time for patients in Group A, and symptomatic treatment with drugs would be provided for all other patients (Figure 1).

Group A, idiopathic Parkinson's disease (PD) and ready for surgery; Group B, idiopathic PD and potentially ready for surgery in the future; Group C, idiopathic PD but never applicable for surgery; Group D, neither idiopathic PD nor a candidate for deep brain stimulation surgery.

FLASQ-PD, Florida Surgical Questionnaire for Parkinson's Disease; UPDRS-III, Unified Parkinson Disease Rating Scale III.

### 2.4. Data Analysis

Cronbach's alpha of the Chinese FLASQ-PD was calculated using SPSS 26.0 (IBM, Armonk, NY, USA) to evaluate internal consistency in all patient samples. Mean group comparisons were, respectively, evaluated in different diagnostic and patient categories using nonparametric tests (Kruskal–Wallis test) and pairwise comparisons. Pearson's correlation coefficient was calculated to find the potential correlation between the FLASQ-PD and UPDRS-III scores. All significant levels were set at 0.05, excluding alpha levels in multiple tests (Bonferroni method), which were adjusted accordingly. Youden's index was calculated to optimize both sensitivity and specificity of the thresholds to define groups of patients where surgery was applicable or was not applicable, respectively.

## 3. Results

### 3.1. General Patient Information

In total, 146 patients comprising 115 with PD, 25 with Parkinsonism, and six with multiple system atrophy (MSA) were consecutively included in the Departments of Functional Neurosurgery and Neurology from June 4th to December 31st, 2019 (Table 1). Patients were divided into predefined categories: 66 patients into Group A, 45 into Group B, four into Group C, and 31 into Group D (Tables 2 and 3). Internal consistency of the Chinese FLASQ-PD was roughly adequate (Cronbach's alpha = 0.664).

### 3.2. Comparisons among Patient Groups

There was a significant difference in mean total scores of the Chinese FLASQ-PD among the diagnostic groups (*H* = 37.450, *p* ≤ 0.001). Furthermore, all subscores significantly differed among the three disease groups (H value of Section B = 95.470, *p* ≤ 0.001; H value of Section C = 18.405, *p* ≤ 0.001; H value of Section D = 10.709, *p*=0.005; H value of Section E = 17.906, *p* ≤ 0.001). With the exception of Section D (*p* of parkinsonism versus MSA = 0.678, *p* of parkinsonism versus PD = 0.003; *p* of MSA vs. PD = 1.000), mean subscores in Sections B, C, and E of the PD group were higher than in groups of patients diagnosed clinically with parkinsonism or MSA (Table 2).

A significant difference was also found in the mean total scores of the Chinese FLASQ-PD among the surgery-candidacy groups (*H* = 48.352, *p* ≤ 0.001). All subscores also significantly differed among the four groups (H value of Section B = 94.862, *p* ≤ 0.001; H value of Section C = 45.886, *p* ≤ 0.001; H value of Section D = 9.421, *p*=0.024; H value of Section E = 18.812, *p* ≤ 0.001). In Section B, the mean subscore of Group D was significantly lower than the other three groups (adjusted *p* ≤ 0.05), and the mean subscore of Group A in Section C was higher than the other three groups (adjusted *p* ≤ 0.001). However, in Sections D and E and in the total score, the difference (adjusted *p* ≤ 0.05) stemmed from the comparison of Groups A and D and the comparison of Groups B and D (Table 3).

### 3.3. Correlation between Chinese FLASQ-PD and UPDRS-III

Improvements between the off and on medication UPDRS-III scores were obtained only in Group A. The average improvement in UPDRS-III score was 48.0 ± 13.3% in these patients. The drug improvements of UPDRS-III score were mildly correlated with scores of the Chinese FLASQ-PD in Group A patients (Pearson's correlation = 0.399, *p*=0.001).

### 3.4. Thresholds of Chinese FLASQ-PD

Two cut-off points were settled in this study to reflect the appropriateness of surgical candidacy. A score of ≥28 (sensitivity = 0.949 in this patient group) was likely to reflect the probable PD surgical candidacy, and a score of ≤22 (specificity = 0.875 in this patient group) may suggest either advanced PD or other parkinsonian syndrome and, therefore, reflected poor surgical candidacy. Youden's index was 0.824 with such a threshold setting. When the score was between 23 and 27, further evaluation by the professional clinician would help in making an optimal decision on whether the patient should maintain drug treatment or prepare for DBS surgery.

## 4. Discussion

The Chinese version of the FLASQ-PD was initially validated in this study as the internal consistency was comparable to the original version of FLASQ-PD (Cronbach's alpha = 0.664 in this study and 0.69 in the English version). Two cut-off points could be used to divide patients into three groups: “appropriate for DBS surgery,” “not qualified for surgery,” and “further evaluation required.” Both sensitivity and specificity were adequate; therefore, this questionnaire could be a helpful and simple screening tool for Chinese neurologist and physicians to refer patients to a functional neurosurgery unit.

During the past decade, well-known functional neurosurgery centers in China have made progress in PD. Long-term follow-up of PD patients confirmed motor function improvement produced by DBS treatment [15, 16]. New methods have been attempted in PD patients to achieve further improvement of DBS outcomes, such as combined unilateral STN and contralateral GPi DBS developed by Zhang et al. and wearable sensors developed by Wang et al. to measure step angle for quantifying the gait impairment of PD patients [2, 17]. The number of new functional neurosurgery units has gradually increased in China; however, this has given rise to both achievements and problems in recent years. Primarily, the initial question of whether a PD patient is an appropriate candidate for DBS treatment has often challenged clinicians. Whether a patient had idiopathic PD is often more confusing to a neurosurgeon than a neurological physician. A non-PD patient should not be considered for DBS in the first place. Additionally, identifying whether a PD patient was qualified for DBS treatment became more difficult to a neurological physician than a neurosurgeon. The timing of DBS surgery serves as an indicator for the stage of the disease, which should be considered carefully [8].

Therefore, such a screening tool could help clinicians to make decisions in both diagnosis and treatment. However, the results of the questionnaire should be cautiously interpreted because it is neither a diagnostic tool nor a final decision on surgery. Indeed, the comparisons among diagnostic groups did not present unified results in subscores of all sections because this questionnaire could not provide a formal diagnosis. The differences in subscores of surgical candidacy groups came partly from the comparison between non-PD patients and patients who were appropriate for DBS surgery. In other words, a PD patient should undoubtedly undergo comprehensive examinations before receiving DBS surgery to exclude late-stage PD and Parkinsonism.

This study had some limitations. The patients in each diagnosis group were not comparable in number, which may have resulted in decreased testing power. Only the surgery-ready group was evaluated by the UPDRS-III. As a result, the correlation between the improvements in UPDRS-III score and the scores of FLASQ-PD was relatively weak, although the statistical significance was adequate. Further study could explore the potential relationship between the scores of FLASQ-PD and the changes of UPDRS-III before and after DBS treatments. Yet, results from a single-center study are not always adequately representative. Prospective studies should be performed in multiple functional neurosurgery units in China for a more robust result to be generalized nationwide, provided that only three diseases are measured in such studies.

## 5. Conclusions

The Chinese FLASQ-PD was developed and initially validated in this retrospective single-center study. Proper referral of PD patients for DBS treatment according to this provides a PD patient with a second choice of treatment method and also simplifies the process and makes it more efficient for clinicians. However, the FLASQ-PD is never intended to replace a multidisciplinary evaluation with a movement disorders team before the final surgery. Cautious screening with this questionnaire would help in the differential diagnosis of idiopathic PD and parkinsonian symptoms and may suggest possible treatment options for specific patients. Further study would be required to confirm the accuracy of the questionnaire among a larger patient group.

## Figures and Tables

**Table 1:** Patient demographic information.

Disease	PD	Parkinsonism	MSA	Total
N	115	25	6	146
Sex	64M, 51F	8M, 17F	4M, 2F	76M, 70F
Age	65.2 ± 8.9	61.5 ± 10.0	63.8 ± 6.5	64.5 ± 9.0
Disease duration (*y*)	9.3 ± 5.5	3.1 ± 2.2	2.5 ± 1.0	7.9 ± 5.6

Mean ± standard deviation; ^*∗*^*N* = the number of patients with the disease; M = male; F = female; PD = Parkinson's disease; MSA = multiple system atrophy. Parkinsonism meant the patients were not idiopathic PD but presented with parkinsonian symptoms, and who were not yet diagnosed by neurologists as having MSA, progressive supranuclear palsy, Parkinson's disease, or dementia.

**Table 2:** Chinese FLASQ-PD score grouped by diagnosis.

Disease	*N*	Section B	Section C	Section D	Section E	Total
PD	115	8.0 ± 0.1	6.1 ± 1.6	11.3 ± 1.4	2.4 ± 1.6	27.8 ± 3.0
Parkinsonism	25	7.1 ± 0.7	4.8 ± 1.5	10.2 ± 1.6	1.3 ± 1.2	23.5 ± 2.9
MSA	6	6.8 ± 0.8	4.3 ± 1.0	11.0 ± 1.5	0.5 ± 0.8	22.7 ± 2.2

Mean ± standard deviation; ^*∗*^*N* = the number of patients with the disease; Section B = potential contraindications for DBS surgery; Section C = general patient characteristics; Section D = favorable/unfavorable characteristics for DBS surgery; Section E = medication information; PD = Parkinson's disease; MSA = multiple system atrophy.

**Table 3:** Chinese FLASQ-PD score grouped by surgery candidacy.

Surgery candidacy	*N*	Section B	Section C	Section D	Section E	Total
Group A	66	8.0 ± 0.0	6.7 ± 1.3	11.3 ± 1.4	2.6 ± 1.6	28.6 ± 3.0
Group B	45	8.0 ± 0.1	5.5 ± 1.5	11.4 ± 1.4	2.1 ± 1.5	27.0 ± 2.8
Group C	4	8.0 ± 0.0	3.0 ± 0.8	11.3 ± 1.0	2.3 ± 2.1	24.5 ± 1.7
Group D	31	7.1 ± 0.7	4.7 ± 1.4	10.4 ± 1.6	1.1 ± 1.1	23.3 ± 2.8

Mean ± standard deviation; ^*∗*^*N* = the number of patients with the disease; Section B = potential contraindications for DBS surgery; Section C = general patient characteristics; Section D = favorable/unfavorable characteristics for DBS surgery; Section E = medication information; Group A = idiopathic PD and ready for surgery; Group B = idiopathic PD and potentially ready for surgery in the future; Group C = idiopathic PD but never applicable for surgery; Group D = neither idiopathic PD nor a candidate for DBS surgery.

## Data Availability

The data used in the study are available on request.

## Supplementary Materials

Supplementary Materials The Chinese version of the Florida Surgical Questionnaire for Parkinson's Disease was attached.
