# Supplementary material for: Development and Initial Validation of the Chinese Version of the Florida Surgical Questionnaire for Parkinson's Disease
Source: Parkinsons Dis. 2020 Dec 12;2020:8811435. doi: 10.1155/2020/8811435 (PMC7749765; doi:10.1155/2020/8811435)
Supplement: Supplementary Materials — The Chinese version of the Florida Surgical Questionnaire for Parkinson's Disease was attached. [file 8811435.f1.pdf]

帕金森病佛罗里达手术问卷（FLASQ-PD）

英文版 © Okun and Foote 2003

University of Florida McKnight Brain Institute, Department of Neurology

中文版 © 上海交通大学医学院附属瑞金医院神经内科和功能神经外科；

患者姓名：______________________________________

患者电话：______________________________________

患者保险：__________________________________

评估日期：__________________________________

评估者：____________________________________

评估地点：__________________________________

请确保你的病人符合英国伦敦脑库帕金森病诊断标准（Hughs, et. al.）的原发性帕金森病诊断：

A. 原发性帕金森病诊断

诊断 1：是否存在动作迟缓？ **是/否**（请画圈作答）

诊断 2：（如存在请打勾）：

___ 强直（肩膀、腿或颈部僵硬）

___ 4-6 赫兹静止性震颤

___ 不是由原发性视觉、前庭、小脑、本体感受器功能障碍所致姿势不稳定

你的病人是否符合以上至少 2 项？ **是/否**（请画圈作答）

诊断 3：（如存在请打勾）：

___ 单侧起病

___ 存在静止性震颤

___ 进展性疾病

___ 持续的、非对称性，起病侧更重

___ 对左旋多巴反应良好（70-100%）

___ 左旋多巴所致严重运动障碍

___ 左旋多巴有效 5 年及以上

___ 临床病程 5 年及以上

你的病人是否符合以上至少 3 项？ **是/否**（请画圈作答）

（对 3 个问题**均作答“是”**提示诊断原发性帕金森病）

B. 提示为非原发性 PD 的表现

原始反射

1- 红旗 – 存在抓握反射、噘嘴反射、觅食反射或迈尔森征

N/A – 无/不详

存在核上性凝视麻痹

1- 红旗 – 存在核上性凝视麻痹

N/A – 无/不详

存在观念运动性失用

1- 红旗 – 存在观念运动性失用

N/A – 无/不详

存在自主神经功能障碍

1- 红旗 – 发病 1-2 年存在非药物性新发严重直立性低血压、勃起功能障碍或其他自主神经功能紊乱

N/A – 无/不详

存在阔基步态

1- 红旗 – 存在阔基步态

N/A – 无/不详

存在轻度以上痴呆

1- 红旗 – 频繁定向障碍、严重认知困难、严重记忆困难或命名障碍

N/A – 无，不详

存在严重精神病

1- 红旗 – 存在严重药物难治性精神病

N/A – 无，不详

左旋多巴无效治疗史

1- 红旗- 帕金森症明显对左旋多巴治疗无反应，或病人从未服用过左旋多巴，或没有经历完整的左旋多巴治疗方案

N/A – 无，不详

（上述**任何**“红旗标记”可能是手术禁忌症）

C. 病人特征（请圈出最能说明你帕金森病手术候选人特征的答案）：

1. 年龄：

0 - >80

1 – 71-80

2 – 61-70

3 - <61

2.帕金森症状病程：

0 - <3 年

1 – 4-5 年

2 - >5 年

3. 症状波动（剂末现象，在异动症和运动不能间波动）？

0 – 否

1 – 是

4. 异动症

0 – 无

1 - <50%的时间

2 - >50%的时间

5. 肌张力障碍

0 – 无

1 - <50%的时间

2 - >50%的时间

一般病人特征，总分____

D. 有利/不利因素

6. 冻结步态

0 – 在“开”期中最好时，左旋多巴对其无效

1 –在“开”期中最好时，左旋多巴对其有效

NA – 不适用

7. 姿势不稳定性

0 –在“开”期中最好时，左旋多巴对其无效

1 –在“开”期中最好时，左旋多巴对其有效

NA – 不适用

8. 华法令或其他抗凝血疗法

0 – 除了抗血小板治疗，正在服用华法令或其他抗凝血剂

1 – 除了抗血小板治疗，未服用华法令或其他抗凝血剂

9. 认知功能：

0 - 记忆困难或额叶功能缺陷

1 – 无认知功能障碍症状或体征

10. 吞咽功能

0 – 频繁哽噎或误吸

1 - 有时哽噎

2 - 很少哽噎

3 - 无吞咽困难

11. 二便控制

0 – 二便失禁

1-仅尿失禁

2-无失禁

12. 抑郁

0 – 严重抑郁伴自主神经症状

1 – 已治疗，中度抑郁

2 – 轻度抑郁症状

3 – 无抑郁

13. 精神病：

0 – 频繁幻觉

1 – 偶尔幻觉-很可能为药物相关

2 – 无幻觉

有利/不利特征，总分____

E. 药物试验（圈出最佳答案）

14. 左旋多巴疗效：

0 不确定左旋多巴是否有效，或无左旋多巴治疗史

1 – 左旋多巴中度改善症状

2 – 左旋多巴显著改善症状

15. 复合左旋多巴，如息宁（卡比多巴/左旋多巴）、美多芭或等效药物：

0 – 没用过或每天不足 3 次

1 – 每天 3 次

2 – 每天 4 次

3 – 每天大于 4 次

16. 多巴胺受体激动剂试验：

0 – 无试验或每天不足 3 次

1 – 多巴胺受体激动剂每天 3 次

2 – 多巴胺受体激动剂每天 4 次

3 – 多巴胺受体激动剂每天大于 4 次

17. 息宁增效剂试验

0 – 没用过

1 – 托卡朋或恩他卡朋试验

18. 联合息宁或多巴胺受体激动剂等效药物试验

0 – 无试验

1 – 息宁或多巴胺受体激动剂等效药物试验

药物试验分项得分：_____

FLASQ-PD 得分：

A. 符合原发性 PD 诊断标准：是/否

B. 禁忌症（FLAGS），得分：____ （8 分可能，任何红旗标记意味着可能不是良好的手术候选病人）

C. 一般特征，得分____ （10 分可能）

D. 有利/不利特征，得分：____ （14 分可能）

E. 药物试验，得分____ （10 分可能）

量表总分（C+D+E）：____ （34 分可能）

存在难治性震颤： 是/否

是/否（部分患者存在大剂量左旋多巴、或者左旋多巴联合多巴胺受体激动剂、抗胆碱能药物治疗无效的中、重度震颤，可不依赖上述评分做出手术决定）
